# Ischemia and reperfusion injury combined with cisplatin induces immunogenic cell death in lung cancer cells

**DOI:** 10.1038/s41419-022-05176-y

**Published:** 2022-09-03

**Authors:** Shuai Zhang, Yumei Li, Shuqing Liu, Pei Ma, Mengfei Guo, E. Zhou, Limin Duan, Jinshuo Fan, Tingting Liao, Qi Tan, Xuan Wang, Feng Wu, Yang Jin

**Affiliations:** 1 Department of Respiratory and Critical Care Medicine, Key Laboratory of Respiratory Diseases of National Health Commission, Clinical Research Center for major respiratory diseases in Hubei Province, Union Hospital, Tongji Medical College, Huazhong University of Science and Technology, Wuhan, Hubei China; 2 Department of Respiratory and Critical Care Medicine, The Eighth Affiliated Hospital, Sun Yat-sen University, Shenzhen, Guangdong China; 3 State Key Laboratory of Respiratory Disease, The First Affiliated Hospital of Guangzhou Medical University, Guangzhou, China; 4 Department of Critical Care Medicine, Union Hospital, Tongji Medical College, Huazhong University of Science and Technology, Wuhan, Hubei China

**Keywords:** Cancer immunotherapy, Chemotherapy

## Abstract

A first-line chemotherapeutic drug for non-small cell lung cancer (NSCLC), cisplatin (CDDP), fails to induce immunogenic cell death (ICD) because it fails to induce calreticulin (CRT) exposure on the cell surface. We investigated the potential of ischemia and reperfusion injury (I/R) combined with CDDP to induce ICD in lung cancer cells. The in vitro model of I/R, oxygen-glucose deprivation and reperfusion (OGD/R), effectively induced CRT exposure, ATP secretion, high mobility group box 1 (HMGB1) release and eIF2α phosphorylation in both Lewis lung carcinoma (LLC) and A549 cells when combined with CDDP. By using a vaccine assay and coculture with bone marrow-derived dendritic cells (BMDCs), we showed that OGD/R restored the immunogenicity of CDDP by phosphorylating eIF2α and demonstrated that OGD/R + CDDP (O + C) is an ICD inducer. Using the inguinal tumor model, we found that I/R significantly enhanced the tumor-killing effect of CDDP and Mitomycin C, and this effect relied on adaptive antitumor immunity. Consistently, I + C altered the ratio of interferon-gamma-secreting T lymphocytes, thus overcoming the immunosuppressive effect induced by CDDP. In conclusion, our research presents a new combination strategy and indicates that I/R is a potential anticancer immunogenic modality when combined with nonimmunogenic chemotherapy.

## Introduction

For a long time, apoptotic cells were considered to be nonimmunogenic or even tolerogenic. However, Casares et al. showed that doxorubicin may induce immunogenic apoptosis and that caspase activation is required for the immunogenicity of doxorubicin-induced apoptosis [1]. Thus, a new concept of immunogenic cell death (ICD), i.e., a cell death modality that stimulates an immune response against dying cancer cell antigens, emerged [2]. ICD is mainly mediated by surface-exposed calreticulin (ecto-CRT), secreted adenosine 5′-triphosphate (ATP) and released high mobility group protein B1 (HMGB1) [3–6]. In ICD, ecto-CRT acts as the “eat-me” signal, secreted ATP acts as the “find-me” signal, and HMGB1 acts as a strong cytokine and attracts various immune cells [7]. ICD can be induced by various agents, such as cetuximab, photodynamic therapy, radiotherapy, oxaliplatin (OXA) and anthracycline [8–10]. However, only a limited number of chemotherapeutic drugs can induce ICD. Since chemotherapy is the most widely used therapy in advanced cancer patients, it is of great importance to find new ways to restore the immunogenicity of chemotherapeutic drugs that cannot induce ICD.

Like most chemotherapeutic drugs, the first-line chemotherapeutic drug for lung cancer, cisplatin (CDDP), is not able to induce ICD because it cannot induce the exposure of calreticulin (CRT) on the cell surface [11]. Further studies demonstrated that CDDP cannot induce endoplasmic reticulum (ER) stress and thus fails to induce the exposure of CRT on the cell surface [11]. However, previous studies have demonstrated that ER stress inducers can promote the exposure of CRT on the cell surface, thus restoring the immunogenicity of CDDP [11–13]. Therefore, restoring the immunogenicity of CDDP or other nonimmunogenic chemotherapeutic drugs by combination with agents that can promote CRT exposure is considered a promising therapeutic strategy. Ischemia and reperfusion injury (I/R) is a pathological process that occurs when the blood supply to an organ is blocked and reperfusion occurs [14]. I/R is characterized by calcium overload and reactive oxygen species (ROS) generation. The in vitro model oxygen-glucose deprivation and reperfusion (OGD/R) is well used to mimic the in vivo I/R model [15–17]. Previous studies have demonstrated that I/R and OGD/R may induce ER stress and activate the PERK-eIF2α pathway in mouse brain tissue or neuroblastoma N2A cells [18, 19]. ER stress is crucial for ICD and activates danger signaling pathways that help traffic damage-associated molecular patterns (DAMPs) to the extracellular space [7, 13]. We speculated that I/R and its in vitro counterpart OGD/R may restore the immunogenicity of CDDP.

Here, we performed a preclinical study using I/R + CDDP (I + C) in an inguinal subcutaneous Lewis lung carcinoma (LLC) mouse model and tested the tumor-killing effect of this treatment. Using a well-characterized I/R model, we found that I + C significantly inhibited LLC tumor growth and overcame the immunosuppressive effect induced by CDDP. By using an in vitro model of I/R, we demonstrated that OGD/R + CDDP (O + C) is a potential ICD inducer. We further tested the immunogenicity of O + C-treated LLC cells by coculturing these cells with bone marrow-derived dendritic cells (BMDCs). Mechanistically, OGD/R triggers ER stress and the release of DAMPs, which restores the immunogenicity of CDDP. Our study presents a new strategy to restore the immunogenicity of CDDP and activate antitumor immunity during chemotherapy.

Here, we investigated the possible impact of I/R + CDDP (I + C) on cancer immunosurveillance in preclinical models. We determined the effects of I + C and its in vitro model OGD/R + CDDP (O + C) on immunogenic cancer cell stress pathways and their antitumor effects on an inguinal subcutaneous Lewis lung carcinoma (LLC) mouse model. We found that I + C induces immunostimulatory ER stress, hence favoring immune-dependent tumor suppression.

## Results

### Identification of O + C as a candidate ICD inducer

**Fig. 1:**
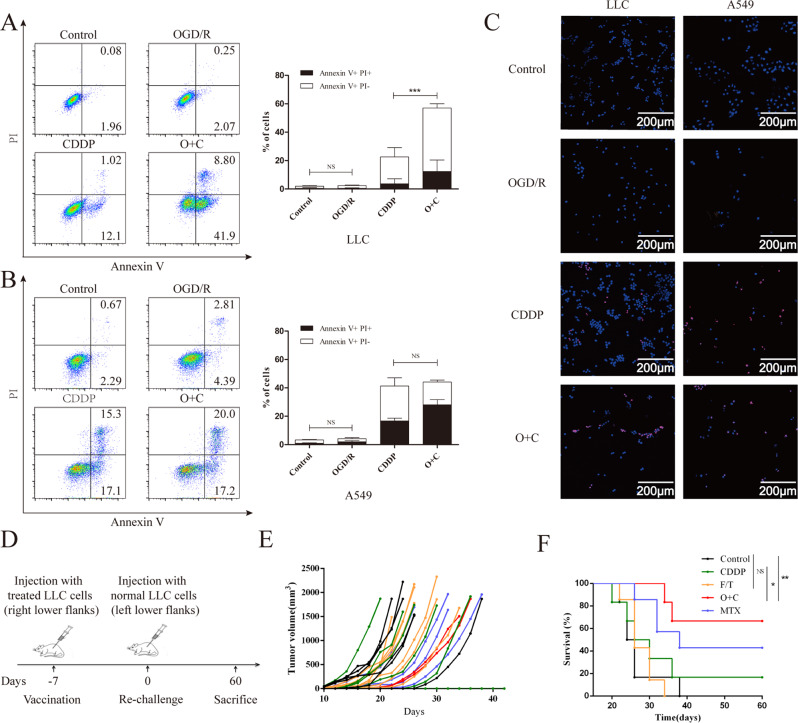
O + C induces immunogenic lung cancer cell death. **A**–**C** LLC and A549 cells were treated with Dimethyl fumarate (DMF) solvent control, OGD/R, CDDP or O + C at the indicated dose for 20 h and then stained with an anti-annexin-V fluorescein isothiocyanate (FITC)-conjugated antibody and propidium iodine (PI) before a subsequent analysis by flow cytometry **A**, **B** or by TUNEL staining **C**. **D**, **E** PBS (*n* = 6), F/T LLC cells (*n* = 7) and LLC cells treated with1 μM MTX (*n* = 7), 100 μM CDDP (*n* = 6), or O + C (*n* = 6), for 20 h and incubated for 4 h without the chemotherapeutic drugs were inoculated into immunocompetent C57BL/6 mice, which were rechallenged 7 days later with live LLC cells **D**. The tumor growth curves **E** and overall survival **F** of C57BL/6 mice after injected with different tumor vaccine are shown. The quantification in A combines data from three independent experiments. The quantification in B combines data from four independent experiments. Data are reported as the mean ± SEM, and statistical analyses were performed with a two-tailed unpaired Student’s t test. The tumor incidence in the vaccination experiments was analyzed by means of the log-rank test. *P < 0.05, **P < 0.01, and ***P < 0.001.

ICD inducers must be cytotoxic [20], so we tested the tumor-killing effect of O + C in vitro. In response to OGD/R, CDDP or O + C, LLC and A549 cells underwent cell death, as indicated by staining with an anti-annexin-V fluorescein isothiocyanate-conjugated antibody and the vital dye propidium iodide. We identified that compared with the untreated cells, the CDDP-treated LLC cells displayed an improved death rate (Fig. 1A), which was further enhanced in the O + C-treated cells. However, OGD/R did not enhance the tumor-killing effect of CDDP on A549 cells (Fig. 1B), which may be related to the inconsistent sensitivity of the two cell lines to ROS and CDDP (Fig. S1). Interestingly, unlike previous studies, we found that OGD/R alone failed to cause tumor cell death in either LLC or A549 cells (Fig. 1A, B). This finding might be related to the use of different hypoxia times and different cell types. The same results were also observed in the terminal deoxynucleotidyl transferase dUTP nick-end labeling (TUNEL) assay (Fig. 1C).

The gold standard of ICD induction is animal vaccination experiments [21]. In a series of experiments involving OGD/R-exposed cancer cells, mice developed tumors at the primary injection site, which was consistent with our results above. In contrast, CDDP-, O + C- and MTX-treated LLC cells failed to form tumors at the primary injection site. After 60 days of observation, we found that C57BL/6 mice injected with the CDDP-treated cell vaccine failed to develop a protective anticancer response, while the O + C- and MTX-treated cells acquired the capacity to stimulate a productive immune response (Fig. 1E, F). Since the tumor death rate of the O + C-treated LLC cells was much higher than that of the CDDP-treated cells, it is possible that the protective effect is due to the death of the tumor cells. Therefore, we also used induced necrosis as a control and found that LLC cells exposed to 3 rounds of freeze/thaw (F/T) did not induce a protective effect against a live LLC cell rechallenge.

**Fig. 2:**
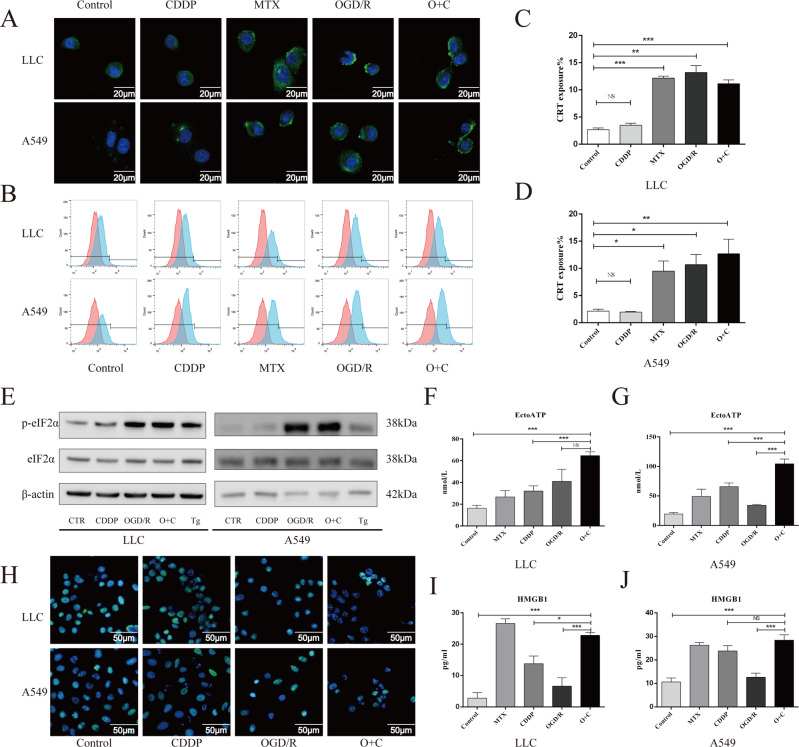
O + C increases the levels of ICD-associated DAMPs. **A**–**D** LLC and A549 cells were treated with DMF solvent control, MTX, CDDP, OGD/R or O + C at the indicated dose for 6 hours, followed by the confocal imaging and cytofluorometric detection of ecto-CRT. **E** LLC and A549 cells were treated with DMF solvent control, CDDP (100 μM), OGD/R, O + C or thapsigargin (Tg, 2 μM) for 30 min, then cells were harvested, and proteins were detected by western blot. **F**, **G** LLC and A549 cells were treated as in A and B for 20 h, and the supernatant was deproteinized and assessed for extracellular ATP by a luciferase-based assay. **H**–**J** LLC and A549 cells were treated as in A and B for 24 h, and then, the representative confocal images show the HMGB1 distribution in cells **H**, and the release of HMGB1 into the supernatant was assessed by an HMGB1-specific ELISA **I**, **J**. The quantification combines data from three independent experiments. Data are reported as the mean ± SEM, and statistical analyses were performed with one-way ANOVA followed by the Dunnett post hoc test. *P < 0.05, **P < 0.01, and ***P < 0.001.

Ecto-CRT, secreted ATP and released HMGB1 are vital for cancer cell ICD. Accordingly, similar to the results of previous studies [11, 12, 22], immunofluorescence staining and confocal microscopic examination (Fig. 2A) or flow cytometry analysis (Fig. 2B–D) indicated that CDDP was unable to induce CRT exposure in LLC and A549 cells in vitro, but this defect in CRT exposure was corrected in the presence of OGD/R. Notably, although OGD/R and O + C induced CRT exposure, they did not upregulate CRT expression in LLC and A549 cells (Fig. S2A–C). A previous study demonstrated that eIF2α phosphorylation correlated with CRT exposure and constitutes a pathognomonic characteristic of ICD [23]. We evaluated eIF2α phosphorylation by performing Western blotting in LLC and A549 cells and found that OGD/R and O + C effectively triggered eIF2α phosphorylation (Fig. 2E, Fig. S2D, E). Moreover, although CDDP triggered ATP secretion in both LLC and A549 cells, this secretion was further enhanced in the presence of OGD/R (Fig. 2F, G). Similar to previous studies, our study found that CDDP exhibited loss of the nucleic HMGB1 fluorescent signal and increased the release of HMGB1. When combined with OGD/R, enhanced loss of nucleic HMGB1 fluorescent signal and HMGB1 secretion was observed in LLC cells but not in A549 cells (Fig. 2H–J, Fig. S2F). This result might be associated with the tumor death rate since the tumor death rates of the CDDP- and O + C-treated A549 cells were not significantly different (Fig. 1B). In conclusion, our results demonstrated that O + C was effective in inducing CRT exposure, ATP secretion and HMGB1 release in both LLC and A549 cells.

### O + C-treated cells are phagocytized and induce DC maturation

**Fig. 3:**
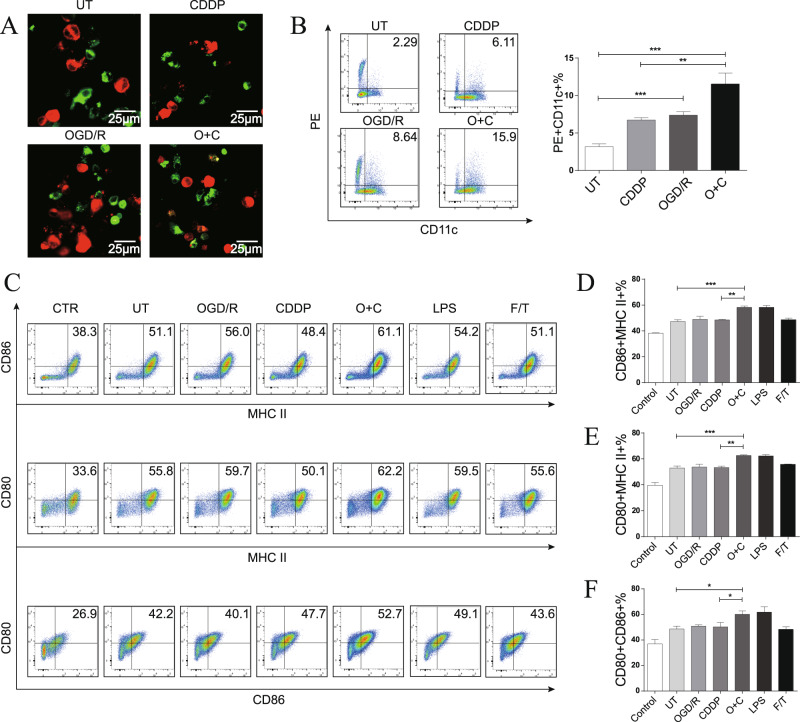
O + C-treated LLC cells are phagocytized and induce bone marrow-derived dendritic cell maturation. **A** Representative confocal images show the uptake of DiI-labeled cellular material by DiO-labeled BMDCs. **B** Representative flow cytometry dot plots show the uptake of DiI-labeled cellular material by BMDCs. Untreated, CDDP-treated, OGD/R-treated and O + C-treated cells were incubated with the BMDCs for 4 h. The phagocytosis assay shows effective uptake of the O + C-treated LLC cells by the BMDCs. **C**–**F** Representative dot plots show the gating and percentages of CD11c + MHC-II + CD80 + , CD11c + MHC-II + CD86 + and CD11c + CD80 + CD86 + BMDCs that were cocultured at a ratio of 1:4 with OGD/R-treated cells, CDDP-treated cells, O + C-treated cells, or F/T-treated necrotic LLC cells for 20 h. LPS was used as a positive control. The quantification of B combines data from three independent experiments, and the quantification of E to F combines data from six independent experiments. Data are reported as the mean ± SEM, and statistical analyses were performed with one-way ANOVA followed by the Dunnett post hoc test. *P < 0.05, **P < 0.01, and ***P < 0.001.

To further assess the immunogenic properties of O + C-treated tumor cells, we tested the ability of these cells to be phagocytized and alter the maturation status of the DCs. We first examined the uptake of O + C-treated tumor cells by BMDCs in vitro. The Dil-labeled tumor cells (red) were cocultured with DiO-labeled BMDCs (green) at a ratio of 1:4 for 4 h, as shown in Fig. 3A, B, and the O + C-treated cells but not the untreated (UT) cells were effectively engulfed by the BMDCs. The expression of surface costimulatory ligands and MHC-II is vital for antigen presentation and DC migration [24, 25]. We next examined the impact of O + C-treated tumor cells on the phenotypic properties of BMDCs. We found that BMDCs cocultured with the O + C-treated cells at a 1:4 ratio showed upregulated MHC-II + CD80 + , MHCII + CD86 + and CD80 + CD86 + cells (Fig. 3C–F). Together, these results indicated that O + C-treated tumor cells were more likely to be captured by the immune system than other cells and were potent inducers of BMDC phenotypic maturation.

### O + C-induced ICD relies on the eIF2α/CRT axis

**Fig. 4:**
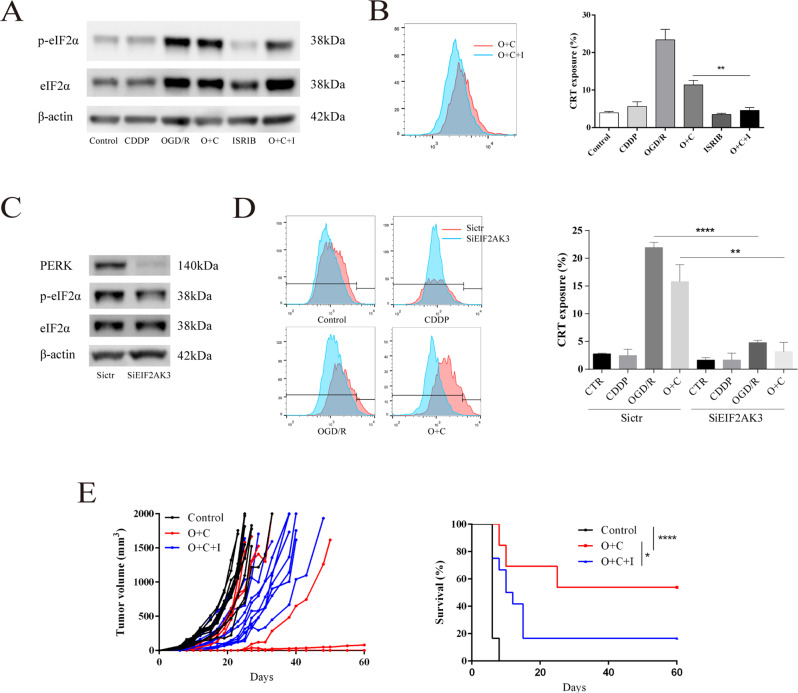
O + C induced ICD relies on eIF2α phosphorylation. **A** LLC cells were treated with OGD for 7.5 hours, followed by 5 nM of ISRIB for 30 minutes before reperfusion, during reperfusion, 5 nM of ISRIB together with 100 μM of CDDP were added for another 30 min, then cells were harvested, and proteins were detected by western blot. **B** LLC cells were treated with OGD for 7.5 hours, followed by 5 nM of ISRIB for 30 minutes before reperfusion, during reperfusion, 5 nM of ISRIB together with 100 μM of CDDP were added for 6 h, then cells were harvested and the CRT exposure were tested by flow cytometry, *n* = 6 biological replicates, the quantification of combines data from three independent experiments. **C** Western blot of EIF2AK3 knockdown LLC cells. **D** EIF2AK3 knockdown LLC cells were treated with CDDP, OGD 5 h/R or O + C at the indicated dose for 6 hours, followed by the cytofluorometric detection of ecto-CRT, *n* = 4 biological replicates, three independent experiments were repeated. **E** PBS (*n* = 12) and LLC cells treated with O + C (*n* = 13) and O + C + I (*n* = 12) for 20 h and incubated for 4 h without the chemotherapeutic drugs were inoculated into immunocompetent C57BL/6 mice, which were rechallenged 7 days later with live LLC cells. The tumor growth curves and tumor free survival of C57BL/6 mice after injected with different tumor vaccines are shown. Data are reported as the mean ± SEM, and statistical analyses were performed with two-tailed unpaired Student’s t test **D** and one-way ANOVA followed by the Dunnett post hoc test **B**. The tumor incidence in the vaccination experiments was analyzed by means of the log-rank test. *P < 0.05, **P < 0.01, ***P < 0.001 and ****P < 0.0001.

Next, we tested whether O + C-induced CRT exposure relies on eIF2α phosphorylation. ISRIB, a potent inhibitor of the integrated stress response, was reported to reverse the effects of eIF2α phosphorylation [26]. We found that when 5 nM ISRIB was added 30 minutes before and during reperfusion, O + C-induced eIF2α phosphorylation was significantly decreased (Fig. 4A). The presence of ISRIB also inhibited O + C-induced CRT exposure (Fig. 4B) and partially abolished the protective antitumor immune response of the O + C-treated tumor vaccine (Fig. 4E). Moreover, the knockdown of EIF2AK3 in LLC cells reduced O + C-induced CRT exposure (Fig. 4C, D). These results indicated that eIF2α phosphorylation plays a critical role in O + C-induced ICD.

### I + C but not I/R suppressed mouse inguinal tumor growth

**Fig. 5:**
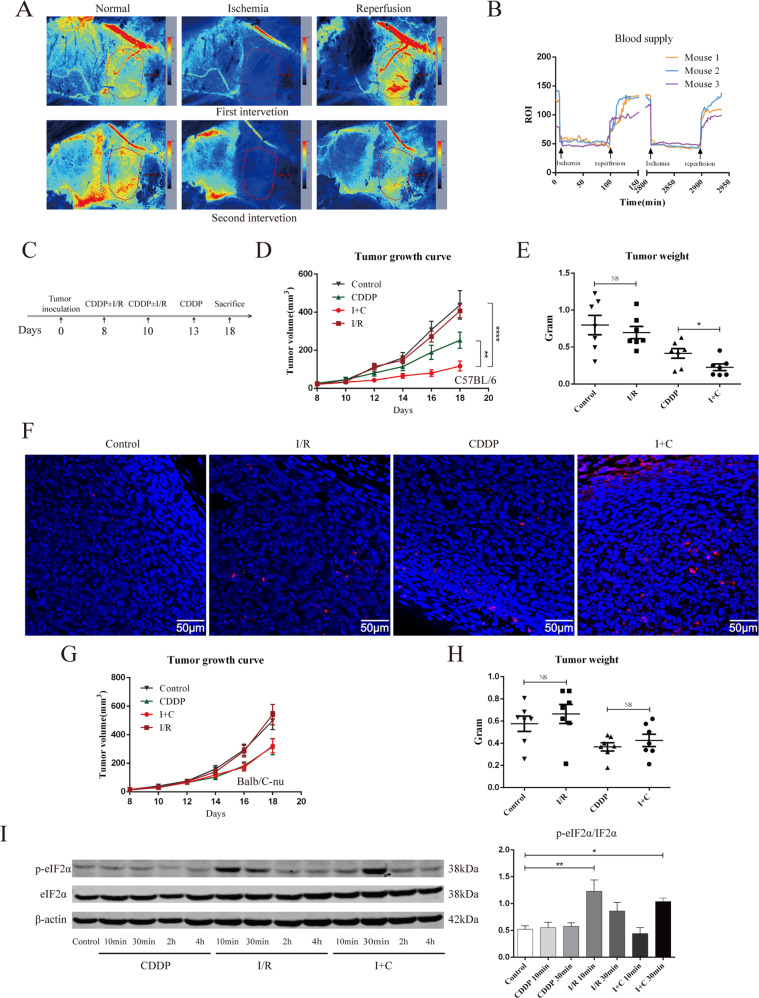
I + C inhibits tumor growth in immunocompetent mice. **A** Laser speckle contrast imaging was used to assess the inguinal tumor blood supply before and during I/R. **B** Laser signal values of the ROI are graphically presented and show the progression from pre-ischemia through reperfusion of the blood supply. Data were recorded every 2 min after clamping the superficial abdominal artery. **C** A total of 5 × 10^5^ cells were inoculated subcutaneously into the right groin of C57BL/6 or Balb/c-nu mice. On days 8 and 10, the right superficial abdominal artery of the mice was blocked for 90 min and then allowed to reperfuse. On days 8, 10 and 13, 3 mg/kg CDDP was injected intraperitoneally. *N* = 7 for each group. On day 18, the mice were sacrificed, the tumor and spleen were collected for further testing. **D**, **E** The tumor growth curve **D** and plot of the tumor weight **E** of the C57BL/6 mice after treated with sham surgery, I/R, CDDP, or I + C are shown. **F** Representative images of TUNEL staining of tumors in C57bl/C mice treated as in A. **G**, **H** The tumor growth curve **G** and plot of the tumor weight **H** of athymic nude mouse Balb/c-nu after being treated as in D and E are shown. *N* = 7 for each group. **I** Mice were treated as in C, after reperfusion for 10 min, 30 min, 2 h, and 4 h, the tumor were removed and proteins were detected by western blot. Data are reported as the mean ± SEM, and statistical analyses were performed with unpaired Student’s t test **E**, **H**, one-way ANOVA followed by the Dunnett post hoc test **I**, and two-way ANOVA **D**. *P < 0.05, **P < 0.01 and ****P < 0.0001.

In our pre-experiment, we tested a variety of tumor models and found that an inguinal tumor model was the ideal model to test the impact of I/R on subcutaneous tumors. Using LSCI, we found that once the superficial abdominal artery was blocked, the blood supply of the inguinal tumor decreased significantly (Fig. 5A, B). The superficial abdominal artery is the main blood supply artery of inguinal tumors. We tested different ischemic times and found that 90 minutes was the most ideal ischemic time for in vivo I/R research. As shown in Fig. S3, after clamping the superficial abdominal artery for 6 hours or 12 hours, no blood reperfusion was observed after removing the vascular clamp. After blocking blood flow for 90 min, the tumor was allowed to reperfuse. We found that the tumor blood supply increased significantly once the vascular clamp was removed. After reperfusion for 30 min, the blood supply could be restored to its previous state or an even higher level in the first intervention (Fig. 5B).

Using the inguinal tumor model, we found that ischemia for 90 min followed by reperfusion could not inhibit LLC tumor growth. Hypoxia can promote tumor invasion [27]. We tested whether I/R can promote tumor recurrence after surgery. As shown in Fig. S4B, completely blocking the tumor’s blood supply before surgery significantly promoted tumor recurrence, while I/R alone did not promote tumor recurrence. Subsequently, we evaluated the capacity of I/R to improve the response to chemotherapy of established tumors growing in immunocompetent or immunodeficient mice. In conditions in which systemic treatment with CDDP or MitoC alone had minor effects on tumor growth, the combination therapy of I + C or I/R + MitoC resulted in a significant therapeutic benefit (Fig. 5D, E, Fig. S4C–F). The TUNEL assay also showed a higher death rate of I + C-treated tumor tissues (Fig. 5F). This effect was observed only when tumors were grown in immunocompetent mice; it was lost when the tumors proliferated in athymic (Balb/c-nu) mice (Fig. 5G, H, Fig. S4G). Previous studies have demonstrated that I/R may induce ER stress and trigger eIF2α phosphorylation [17, 18], and we also tested whether IR can trigger eIF2α phosphorylation in vivo. As shown in Fig. S5I, I/R significantly triggered eIF2α phosphorylation after reperfusion for 10 minutes, while I + C triggered eIF2α phosphorylation after reperfusion for 30 minutes. These results demonstrated that I/R may enhance the tumor-killing effect of CDDP and underscore the essential contribution of the immune system to the therapeutic activity of I + C.

### The tumor-killing effect of I + C relies on antitumor immunity

**Fig. 6:**
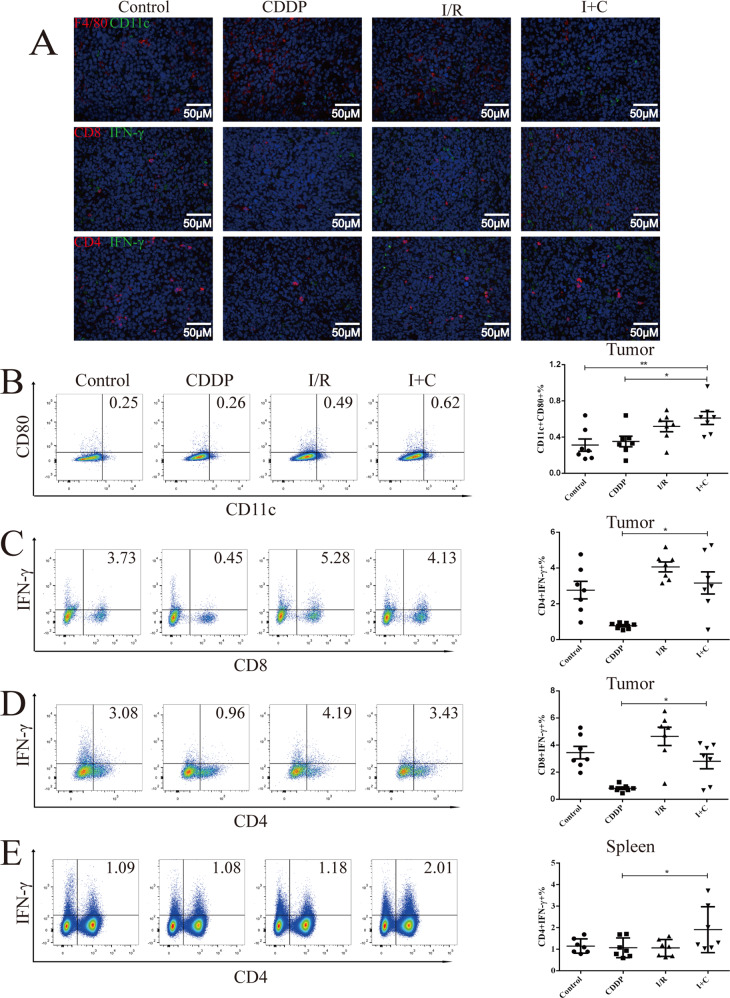
Immune cell subsets of the tumor and spleen in mice. **A** Representative immunofluorescence images show the expression of CD8 (red), IFN-γ (greed), CD4 (red), F4/80 (red) and CD11c (green) in LLC tumors after different treatment. F4/80-CD80 + CD11c + cells **B**, IFN-γ + CD8 + T cells **C**, IFN-γ + CD4 + T cells **D** in the tumor were determined after gating on the live CD45 + cells. IFNγ + CD4 + T cells **E** in the spleen were determined after gating on the live cells. *N* = 7 for each group, each symbol represents a single mouse. Data are reported as the mean ± SEM, and statistical analyses were performed with one-way ANOVA followed by the Dunnett post hoc test **B**, **E**, Kruskal-Wallis followed by the Dunn post hoc test **C**, **D**. *P < 0.05 and **P < 0.01.

To address whether dual therapy could activate local and systemic antitumor immunity, we tested the percentage of T cells, dendritic cells, macrophages, natural killer (NK) cells and myeloid-derived suppressor cells (MDSCs) in tumor-bearing mice receiving different treatments. We found that LLC cancers treated with I + C exhibited a significant increase in the local presence of activated DCs expressing CD11c and CD80 (Fig. 6A, B, Fig. S5A) but no change in the local presence of classic macrophages (M1) (Fig. S5B), alternative macrophages (M2) (Fig. S5C), activated NK cells (Fig. S5D), activated NKT cells (Fig. S5E), or MDSCs (Fig. S5F). In addition, we found that the frequencies of tumor-infiltrating IFN-γ + CD4 + T cells and IFN-γ + CD8 + T cells decreased significantly after treatment with CDDP, but when CDDP was combined with I/R, the frequencies of these cells increased significantly (Fig. 6C, D, Fig. S5A). In the spleen, we found that CDDP did not affect the frequencies of IFN-γ + CD4 + T cells, but I + C led to an increase in the frequencies of these cells compared with those of the control group (Fig. 6E). We also found that compared with the control, I + C decreased the frequency of MDSCs in the spleen (Fig. S5G), but no changes in IFN-γ + CD8 + T cells were observed in the spleen (Fig. S5H). Altogether, these results indicate that I/R can be combined with CDDP to reverse the immune suppressive effect caused by CDDP in an LLC lung cancer model.

## Discussion

ICD inducers have shown promising antitumor effects in animal experiments and clinical studies [28]. Clinical data have shown that TILs are strong positive prognostic factors in human colorectal and breast cancer patients treated with anthracyclines and oxaliplatin, respectively [29–31]. However, only a limited number of chemotherapeutic drugs can induce ICD because most chemical agents fail to trigger the release of key molecules. Moreover, rather than activating antitumor immunity, chemotherapy in cancer patients always induces myelosuppression and therefore suppresses antitumor immunity. Under these circumstances, restoring the immunogenicity of these chemotherapeutic drugs by combination therapy seems to be a promising method. Indeed, it has been reported that CGs restore the immunogenicity of CDDP and increase the overall survival of cancer patients treated with nonimmunogenic chemotherapy but do not enhance the survival of patients treated with immunogenic chemotherapy. Therefore, restoring the immunogenicity of nonimmunogenic chemical agents by combination therapy is of great importance.

According to the guidelines for ICD, a potential ICD inducer must be able to (1) induce tumor cell death, (2) promote the exposure of CRT on the cell surface, (3) increase ATP secretion, and (4) promote the release of HMGB1 [21]. In our experiment, we found that O + C satisfied all four of these conditions in LLC and A549 cells, indicating that O + C is a candidate effective ICD inducer. An immune response will ensue only when DAMPs are correctly emitted by dying cancer cells and perceived by DCs [5]. Our experiment demonstrated that O + C-treated LLC cells altered the maturation status of BMDCs. We also found that the O + C-treated cells, not the OGD/R-treated or CDDP-treated cells, were effectively engulfed by the BMDCs. Moreover, the tumor vaccine assay showed that the O + C-treated tumor vaccine protected mice from tumor rechallenge. These results indicated that O + C caused bona fide ICD. Mechanistically, O + C triggers eIF2α phosphorylation, which results in CRT exposure. Pharmacological or genetic inhibition of eIF2α phosphorylation impaired O + C-induced CRT exposure. In addition, pharmacological inhibition of eIF2α phosphorylation suppressed the protective effect of the O + C-treated tumor vaccine. These results support our hypothesis and demonstrate that OGD/R restores the immunogenicity of CDDP through eIF2α phosphorylation.

Previous studies have demonstrated that I/R significantly inhibits tumor growth and metastasis [32–34]. However, whether I/R inhibits the tumor growth of lung cancer or activates antitumor immunity is still unknown. To test the tumor-killing effect of I/R on the LLC tumor model, we constructed an inguinal xenograft tumor model. By using LSCI, we identified that the blood supply of this tumor model was mainly supplied by the superficial abdominal artery. This finding provided us with a key model to study the impact of I + C on tumors in a highly standardized way. Indeed, we found that the tumor blood supply was significantly decreased after clamping the superficial abdominal artery. During reperfusion, the blood supply of the tumor increased significantly and could return to the initial state within 30 min. These results showed that the inguinal tumor model truly suffered from I/R. These findings also allowed us to rule out the effects of differences in the tumor distribution of CDDP.

By using an in vivo model, our study showed that compared with CDDP, the I + C combination significantly reduced the tumor burden, while I/R alone failed to inhibit tumor growth. This effect was lost when the tumors proliferated in athymic (Balb/c-nu) mice, indicating that the tumor suppressive effect of I + C can be attributed to the adaptive immune system. The cellular components in the tumor showed that I + C increased the frequency of mature dendritic cells. Further analysis also showed that CDDP treatment alone lowered the frequencies of effector T cells (including IFN-γ + CD4 + T cells and IFN-γ + CD8 + T cells), while I + C restored the frequencies of tumor-infiltrating effector T cells, suggesting that the combination therapy reversed the suppression of tumor-specific T cells induced by CDDP. In the spleen, we found that CDDP did not suppress the systemic effector T cells, but I + C showed a trend of increasing the population size of effector T cells, especially that of the IFN-γ + CD4 + T cells. Although the dominant focus of cancer immunotherapy is CD8 + T cells, the role of helper T cells is gaining increasing attention [35, 36]. A recent study demonstrated that the peripheral CD4 + T-cell subset is important for new tumor protection and is associated with a favorable response to immunotherapy in melanoma patients [37]. Our results showed that I + C could activate adaptive antitumor immunity in both the tumor microenvironment (TME) and peripheral tissue.

The potential clinical use for a new kind of therapeutic strategy is the most important. Jia et al. have developed a technology that uses micromagnets to induce aggregation of magnetic nanoparticles to reversibly occlude blood flow in microvessels [38]. This allows induction of I/R in a specific cortical region and makes it possible to apply I/R to different tumor treatments. Our study is the first step, and some other issues should be investigated before clinical use: 1. the sensitivity of different tumor models to I/R plus chemotherapy and 2. the size of magnetic nanoparticles used for reversibly occluding blood flow in tumor microvessels.

In conclusion, we found that I + C significantly inhibited LLC tumor growth and overcame the immunosuppressive effect induced by CDDP. Mechanistically, I/R triggers eIF2α phosphorylation and the release of DAMPs, which restores the immunogenicity of CDDP. Our study presents a new combination therapeutic strategy to restore the immunogenicity of CDDP and provides a theoretical basis for further translational medical research.

## Materials and methods

### Animals and cell culture

Six- to eight-week-old male or female C57BL/6, Balb/c and Balb/c-nu mice were purchased from Beijing Vital River Laboratory Animal Technology Co., Ltd. All mice were maintained in a specific pathogen-free (SPF) environment with a 12 h light-dark cycle at 21 °C to 23 °C and 40% to 60% humidity in the Animal Center of Huazhong University of Science and Technology (HUST), China. All animal experiments were approved by the Animal Care Committee of Tongji Medical College, HuaZhong University of Science and Technology.

The LLC cells and A549 human lung carcinoma cells used in our experiments were purchased from the China Center for Type Culture Collection (Wuhan, China). The LLC cells were cultured in Dulbecco’s modified Eagle’s medium (DMEM), while the A549 cells were cultured in RPMI 1640 medium. All media were supplemented with 10% fetal bovine serum (FBS), 2 mM L-glutamine, 1 mM pyruvate, 100 U/ml penicillin, and 100 μg/ml streptomycin at 37 °C in 5% CO_2_-95% air.

### OGD/R

OGD/R treatment of cells was previously described. 17 Glucose-free DMEM (Gibco) and glucose-free RPMI 1640 medium (Gibco) were pretreated in a hypoxic chamber (Billups-Rothenberg) at 95% N_2_ and 5% CO_2_ overnight. During oxygen-glucose deprivation, tumor cells were washed with phosphate-buffered saline (PBS) twice and then cultured in the pretreated glucose-free DMEM or RPMI 1640 medium and transferred to the hypoxic chamber for 8 h (for negative control and EIF2AK3 knockdown LLC cells, the time reduced to 6 hours since it’s more sensitive to oxygen-glucose deprivation) at 37 °C and 5% CO_2_−95% N_2_. The oxygen level was detected by a Nuvair O_2_ Quickstick and kept below 0.3%. During reperfusion, the glucose-free DMEM or RPMI medium was removed, and the cells were refed with a complete medium with (OGD/R + CDDP) 100 μM CDDP or DMF solvent (OGD/R) and incubated at 37 °C in 5% CO_2_−95% air.

### Freeze/thaw (F/T) procedure

F/T LLC lysates were generated by subjecting cells to three F/T cycles. For each F/T cycle, cryotubes containing LLC cells were transferred into a liquid nitrogen tank (−196 °C) for 5 minutes followed by being immersed in a 37 °C water bath for 6 min to thaw.

### Quantification of cell death by flow cytometry

Cell death was assessed by annexin V-fluorescein isothiocyanate/propidium iodide (PI) staining (BD Biosciences). Briefly, untreated or treated LLC and A549 cells were collected, after being washed in cold PBS once, the cells were resuspended in 1× binding buffer [10 mM HEPES (pH 7.4), 0.14 M NaCl and 2.5 mM CaCl_2_]. Then, an anti-annexin V fluorescein isothiocyanate-conjugated antibody was added to each sample and incubated at 4 °C. Ten minutes later, PI was added to each sample and incubated for another 10 min at 4 °C. All samples were tested by flow cytometry within 30 min.

### In vivo antitumor vaccination

A total of 3 × 10^6 ^F/T LLC cells or 3 × 10^6 ^F/T LLC cells were treated with 100 μM CDDP, 1 μM methotrexate (MTX), OGD/R, O + C for 20 h followed by a 4 h incubation without the chemotherapeutic drugs were injected subcutaneously into the lower flank of 6- to 8-week-old male or female C57BL/6 mice. For O + C + ISRIB tumor vaccine experiment, LLC cells were treated with OGD for 7.5 h, followed by DMSO solvent or 5 nM of ISRIB for 30 min before reperfusion, during reperfusion, 100 μM of CDDP or 5 nM of ISRIB together with 100 μM of CDDP were added for 20 h followed by a 4 h incubation without the chemotherapeutic drugs were injected subcutaneously into the lower flank of 6- to 8-week-old male or female C57BL/6 mice. Seven days later, 100 µl of LLC suspension containing 5 × 10^5^ cells were inoculated into the opposite flank.

### Analysis of ecto-CRT by flow cytometry

As previously reported [39], 2 × 10^5^ LLC and A549 cells were cultured in the presence of DMF solvent, OGD/R, O + C, CDDP or MTX for 6 h, then the cells were harvested, washed twice with cold PBS and fixed in 0.25% paraformaldehyde (PFA) in PBS for 5 min. After two more washes in cold PBS, some cells were incubated for 30 min with an Alexa Fluor® 647-conjugated monoclonal anti-CRT antibody (ab196159, Abcam) diluted in cold blocking buffer (2% FBS in PBS with NaN3). Some cells were incubated for 30 min with a primary anti-CRT antibody (ab2907, Abcam) diluted in cold blocking buffer (2% FBS in PBS with NaN3), washed and incubated with an Alexa Fluor® 488-conjugated monoclonal secondary antibody (ab150077, Abcam) in cold blocking buffer for 30 min. After two more washes in the incubation buffer, 7-aminoactinomycin D (7-AAD) was added to each sample and incubated in the dark for another 10 min at 4 °C. Each sample was then analyzed by flow cytometry to identify ecto-CRT. Samples that were not incubated with the primary antibody were used as isotype controls, and the percentage of CRT exposure of the stained cells was determined according to isotype control after gating on the 7-AAD-negative cells.

### Immunofluorescence

LLC and A549 cells were cultured in the presence of DMF solvent, OGD/R, O + C, CDDP, or MTX for 6 h, and fixed in 4% PFA in PBS for 30 min. After washed with cold PBS twice, the cells were stained with a primary anti-CRT antibody or anti-HMGB1 antibody (ab18256, Abcam) for 30 min followed by incubated with an Alexa Fluor® 488-conjugated monoclonal secondary antibody for 30 min. After two more washes with cold PBS, the cells were mounted on slides with Fluoromount-G, including DAPI.

### ATP assays

LLC and A549 cells were treated with DMF solvent, CDDP, MTX, OGD/R, or O + C for 24 h. Then, the cell supernatants were centrifuged at 400 × *g* for 5 min followed by centrifugation at 2000 × g for 10 min at 4 °C. Next, the supernatants were collected and deproteinized by using perchloric acid and potassium hydroxide. An ATP Colorimetric/Fluorometric assay kit (Abcam, ab83355) was used to quantify the extracellular ATP (ecto-ATP) level following the manufacturer’s instructions. Fluorescence was assessed by a FLUOstar OPTIMA multilabel reader (BMG Labtech, Offenburg, Germany).

### Detection of HMGB1 release

LLC and A549 cells were treated with DMF solvent, CDDP, MTX, OGD/R, or O + C for 24 h, and cell supernatants were collected as previously indicated. The HMGB1 level in the cell supernatants was quantified by means of an enzyme-linked immunosorbent assay (HMGB1 ELISA kit II, Shino Test Corporation, Tokyo, Japan) according to the manufacturer’s instructions. Absorbance and chemiluminescence assessed both assessed by a FLUOstar OPTIMA multilabel reader (BMG Labtech, Offenburg, Germany).

### Western blot

Protein extraction was conducted in radioimmunoprecipitation assay (RIPA) buffer in the presence of phosphatase and protease inhibitors (Thermo Fisher Scientific) followed by sonication. The protein concentration was measured by BCA Protein Assay Kit (Beyotime) and after adding “sample buffer” (50 mM Tris-HCl, 2% SDS, 10% glycerol and 0.1% bromophenol blue), denatured at 100°C. Proteins were subjected to separation by sodium dodecyl sulfate polyacrylamide gel electrophoresis (SDS-PAGE) following protein transfer on a polyvinylidene difluoride (PVDF) membrane. After 1 h block with 5% skim milk powder/tris-buffered saline (TBS)−0.1% tween (TBST), membranes were incubated overnight at 4°C with the following primary antibodies: Anti-PERK antibody (C33E10, Cell Signaling Technology [CST]), Anti-eIF2α antibody (D7D3, CST) and Anti-p-eIF2α (D9G8, CST). After being washed three time with TBST, HRP-conjugated secondary antibodies were incubated for 1 h at room temperature and detected by ECL (Thermo Fisher Scientific). Beta-actin was used to control equal loading.

### Generation of BMDCs

Dendritic cells (DCs) were prepared from bone marrow precursors as described by Lutz [40]. Briefly, 2 × 10^6^ bone marrow cells were cultured in a bacteriological petri dish (Falcon) containing 10 ml of RPMI 1640 medium supplemented with 10% FBS, 2 mM L-glutamine, 1 mM pyruvate, 100 U/ml penicillin, 100 μg/ml streptomycin and 20 ng/ml rmGM-CSF (PeproTech) at 37 °C in 5% CO_2_-95% air. On day 3, 10 ml of fresh medium was added to the petri dish. On days 6 and 8, half of the culture supernatant was collected and centrifuged at 400 × g for 5 min, while the cell pellet was resuspended in 10 ml of fresh medium containing 20 ng/ml rmGM-CSF and returned to the original dish. On day 9, BMDCs were collected for further study.

### Co-culture of tumor cells and BMDCs

For the phagocytosis experiment, LLC cells were stained with 1,1’-dioctadecyl-3,3,3’,3’-tetramethylindocarbocyanine perchlorate (DiI, Beyotime Biotechnology, Shanghai, China) for 20 min, followed by washing with sterilized PBS twice. The LLC cells were treated with DMF solvent, CDDP, OGD/R, or O + C for 20 h, incubated without the chemotherapeutic drug for 4 h and then cocultured with 3,3´-dioctadecyloxacarbocyanine, perchlorate (DiO)-labeled BMDCs at a ratio of 4:1 for 4 h. For the BMDC maturation experiment, LLC cells were treated as previously indicated, and BMDCs pulsed with treated LLC cells or F/T tumor lysate were cocultured with the LLC cells for a 24 h incubation at a ratio of 4:1. BMDCs pulsed with LPS (1 μg/ml) for 24 h were used as a positive control.

### siRNA knockdown of Eif2ak3

Knockdown of Eif2ak3 was performed using DharmaFECT1 transfection reagent and siGENOME Mouse Eif2ak3 siRNA (Horizon, M-044901-01-0005) or 10 nM siGENOME Non-Targeting siRNA (Horizon, D-001206-13-05) according to the manufacturer’s instructions. LLC cells were treated with non-coding or coding siRNA for 48 h and then seeded at 2 × 10^5^ cells/well in a 6 well plate with fresh culture medium for another 24 h before use.

### Tumor model and in vivo ischemia and reperfusion

One hundred microliters of LLC suspension containing 5 × 10^5^ cells were inoculated subcutaneously into the right groin of C57BL/6 or Balb/c-nu mice. Eight days later, the tumor-bearing mice were anesthetized with an intraperitoneal injection of a sodium pentobarbital solution (1%, 100 mg/kg). After the groin area was shaved and sterilized, the femoral artery of the right hind limb was exposed. The control, CDDP and Mitomycin C (Mito C) groups underwent only a sham surgery. For the mice treated with I/R, the femoral artery was clipped with a microvascular clip. Then, the wound was covered with sterile gauze with saline to keep the wound moist. After the blood supply was blocked for specified lengths of time, the microvascular clips were removed to allow reperfusion. All procedures were performed on electric blankets to keep the mice warm. After the mice were awake, CDDP (3 mg/kg), Mito C (2.5 mg/kg) or PBS was injected intraperitoneally into each mouse. For determination of the therapeutic efficacy in each group, tumor volume, tumor weight and body weight evolution were monitored. The tumor volume was calculated as length × width × width/2.

### Laser speckle contrast imaging (LSCI)

Relative tumor blood flow (rTBF) was tested by LSCI in ischemic and reperfusion-treated mice. The tumor-bearing mice were anesthetized with an intraperitoneal injection of a sodium pentobarbital solution. After the groin area was shaved and sterilized, the tumor tissue was exposed. A monochrome camera was used to determine the inguinal tumor area. Then, a custom LSCI system was used to measure the rTBF in regions of interest (ROI), specifically the inguinal tumor region. The level of perfusion was scaled from blue (low perfusion) to red (high perfusion). The initial rTBF was measured every minute for 6 min, and the rTBF during ischemia and reperfusion was measured every two minutes for 90 and 30 min, respectively. For those mice ischemic at 6 h and 12 h, the rTBF during ischemia was measured at the beginning or the end of ischemia. LSCI was performed with a SIM BFI HR Pro Laser Speckle Blood Flow Imager (SIM Opto-Technology Co.,Ltd, Wuhan, China).

### Terminal deoxynucleotidyl transferase dUTP nick-end labeling (TUNEL) assay

The one-step TUNEL Apoptosis Assay Kit (Beyotime Biotechnology, Shanghai, China) was used to assess cell and tumor tissue apoptosis according to the manufacturer’s instructions.

### Flow cytometric analysis

For the phenotypic analysis of BMDCs, cells were stained with the following surface antibodies: FITC-conjugated anti-CD11c (clone HL3), APC-conjugated anti-CD80 (clone 16-10A1), Alexa Fluor® 700-conjugated anti-CD86 (clone GL1), and BV421-conjugated anti-I-A/I-E (clone M5/ 114.15.2). To test the phagocytic clearance ability of BMDCs, DiI-labeled LLC cells were co-cultured with BMDCs for 4 h, and then the cells were stained with an anti-CD11c antibody. Dead cells were excluded using 7-AAD (BD Biosciences). To examine immune cells in the tumor and spleen, flow cytometric analysis was performed with single-cell suspensions isolated from the tumor and spleen as previously described [41]. To obtain single-cell suspension, spleens were grinded, and filtered through 70 µm cell strainer. Tumors were dissected into small pieces (2-3 mm diameter) and incubated in an PBS solution containing 2% FBS, DNAse I (10 mg/ml), and Collagenase IV (1 mg/ml) (all from Sigma-Aldrich, Gillingham, Dorset, UK) for 1 h at 37 °C, then filtered through 70 µm cell strainer. For surface staining, lymphocytes prepared from the tumor tissue and the spleen were stained with the following surface antibodies: APC-Cy7-conjugated anti-CD45 (clone 30-F11), APC-conjugated anti-CD3 (clone 17A2), FITC-conjugated anti-CD4 (clone GK1.5), BV421-conjugated anti-CD8 (clone 53-6.7), PE-Cy7-conjugated anti-CD69 (clone H1.2F3), BV421-conjugated anti-F4/80 (clone T45-2342), PE-conjugated anti-NK1.1 (clone PK136), FITC-conjugated anti-CD11c (clone HL3), Percp5.5-conjugated anti-CD80 (clone 16-10A1), BV605-conjugated anti-CD206 (clone C068C2), PE-Cy7-conjugated anti-CD11b (clone M1/70) and Percp5.5-conjugated anti-Gr-1 (clone RB6-8C5). For intracellular cytokine staining, lymphocytes prepared from TILs and the spleen were restimulated with phorbol 12-myristate 13-acetate (PMA; 50 ng/mL) and ionomycin (1 mg/mL) in the presence of Brefeldin A (1 mg/mL) (all from Sigma-Aldrich, Gillingham, Dorset, UK) for 4 h and then blocked with anti-CD16/CD32 (clone 2.4G2) antibodies. After surface staining, the cells were treated with a Fix/Perm solution (eBioscience) and stained with a PE-conjugated anti-IFN-γ (clone 554412) antibody. In all stained samples, the dead cells were excluded using the Live/Dead Fixable Dead Cell Staining Kit (BioLegend). All antibodies were purchased from BD Biosciences or Biolegand. Cell acquisition was performed with a BD LSRFortessa X-20 flow cytometer (San Diego, CA, USA). The data were analyzed with FlowJo (TreeStar).

### Statistical analysis

All statistics were two-tailed and a p-value less than 0.05 was considered significant. Shapiro-Wilk test was used to test whether data followed a normal distribution and Levene’s F test was used to test whether data met variance homogeneity. All statistical analyses were performed by using SPSS software (IBM SPSS Statistics 21, Chicago).

## Supplementary information

Supplementary Figures

Supplementary Materials for western blot

Reproducibility checklist

## Data Availability

The data that support the findings of this study are available from the corresponding authors upon reasonable request.
