# Supplementary material for: Ischemia and reperfusion injury combined with cisplatin induces immunogenic cell death in lung cancer cells
Source: Cell Death Dis. 2022 Sep 3;13(9):764. doi: 10.1038/s41419-022-05176-y (PMC9440929; doi:10.1038/s41419-022-05176-y)
Supplement: Supplementary file 1 — Supplementary Figures [file 41419_2022_5176_MOESM1_ESM.docx]

**Supplementary** **Figures**

Fig. S1. A549 cells were less sensitive to ROS.

Fig.S2. O+C did not upregulate the expression of CRT but induced DAMPs release.

Fig. S3. Representative laser speckle contrast images of inguinal tumor tissues before and during I/R.

Fig. S4. I/R combined with non-immunogenic chemotherapeutic drugs inhibit tumor growth.

Fig. S5. Immune cell subsets of the tumor and spleen in mice.


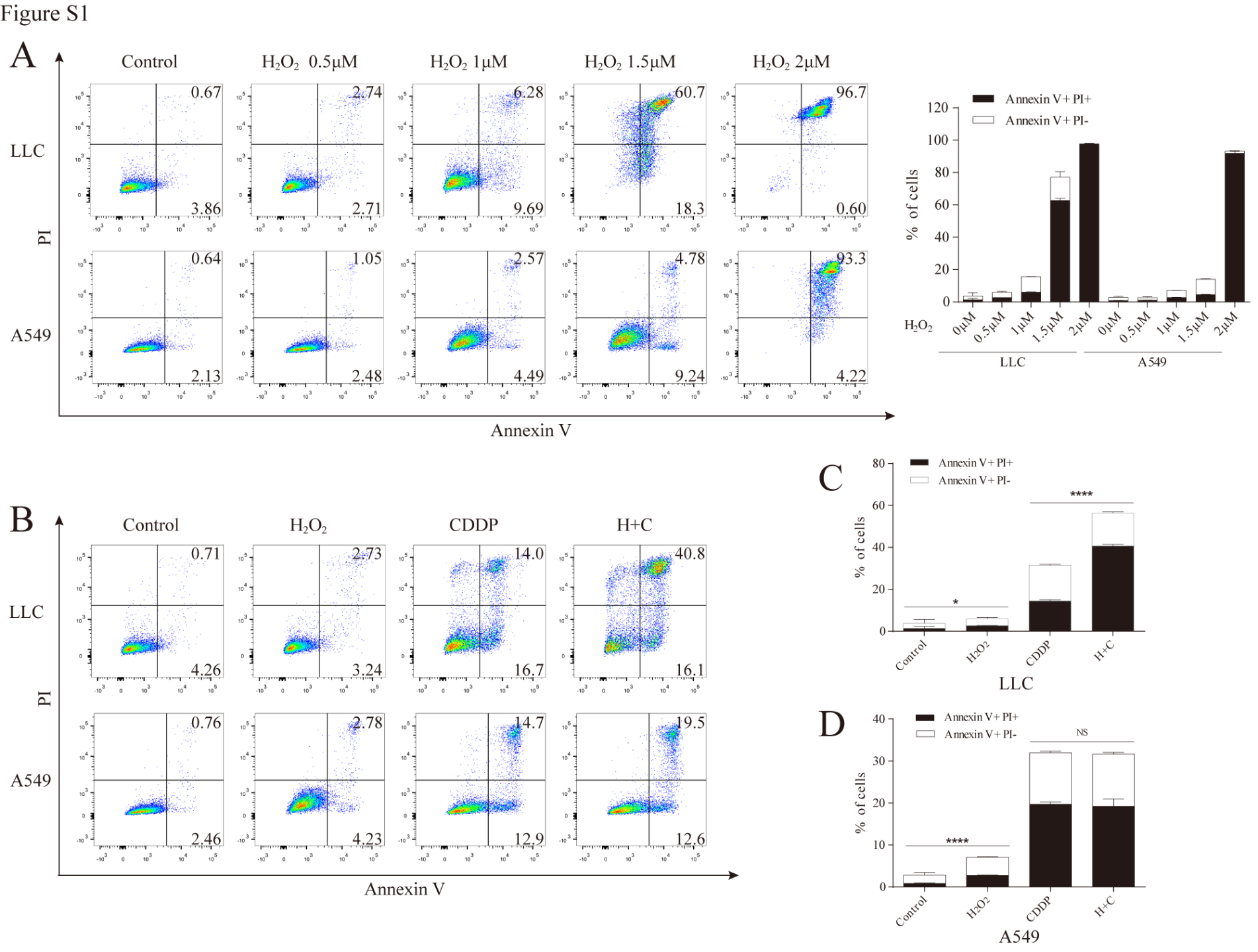


**Figure S1**. A549 cells were less sensitive to H_2_O_2_. **(A)** LLC and A549 cells were treated with H_2_O_2_ at the indicated dose for 20 h, n = 4 biological replicates, three independent experiments were repeated. **(B, C)** LLC cells were treated with 0.5μM H_2_O_2_ with or without 100μM CDDP for 20 h, **(B, D)** A549 cells were treated with 1μM H_2_O_2_ with or without 100μM CDDP for 20 h, and then stained with an anti-annexin-V fluorescein isothiocyanate (APC)-conjugated antibody and propidium iodine (PI) before a subsequent analysis by flow cytometry, n = 4 biological replicates, three independent experiments were repeated. Data are reported as the mean ± SEM, and statistical analyses were performed with one-way ANOVA followed by the Sidak post hoc test. *P < 0.05 and ****P < 0.0001.


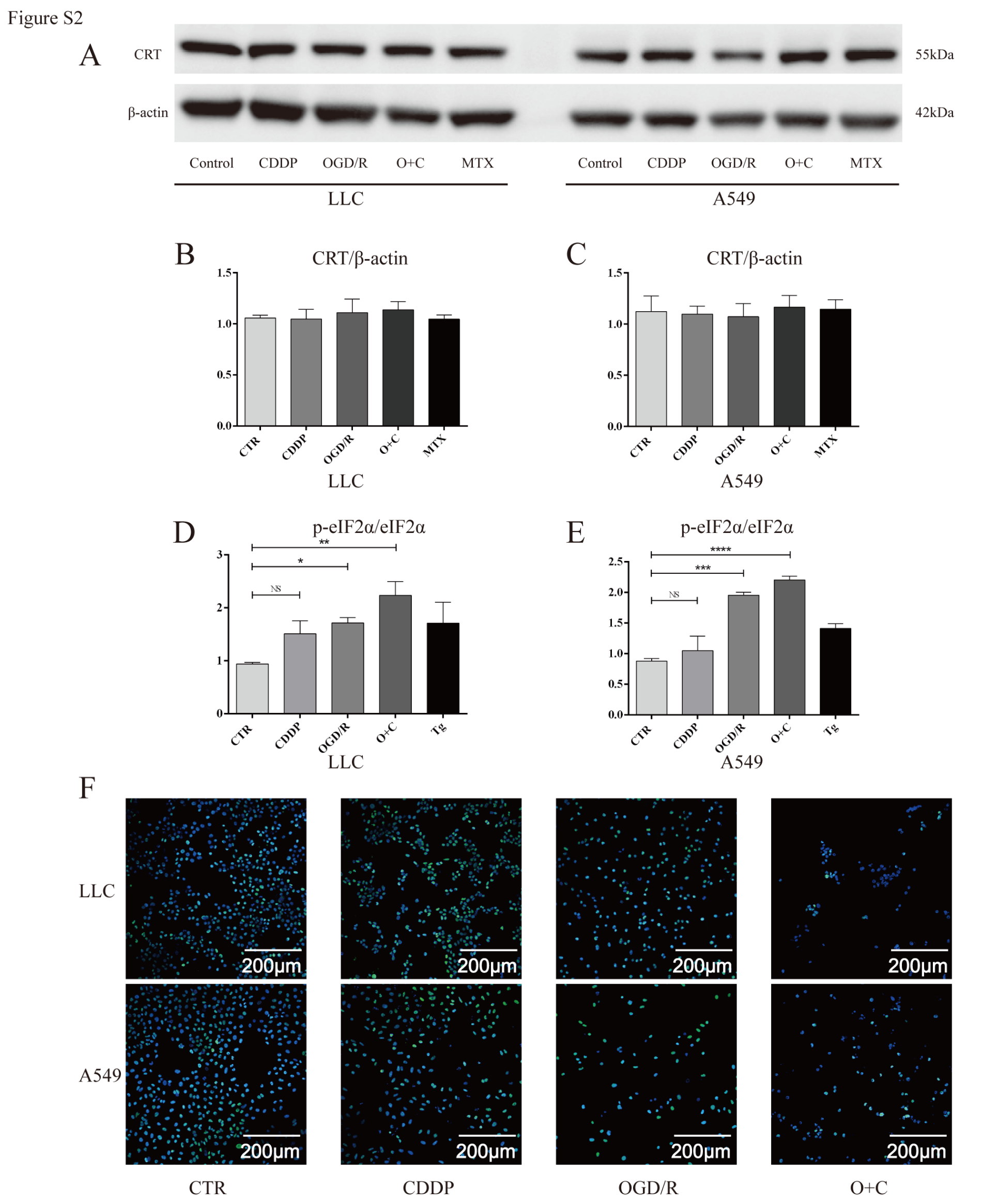


**Figure S2**. O+C did not upregulate the expression of CRT but induced DAMPs release. **(A-C)** LLC and A549 cells were treated with DMF solvent control, CDDP (100μM), OGD/R6h, O+C, or MTX (1μM) for 6 hours, then cells were harvested, and proteins were detected by western blot. Densitometry data of CRT/Actin in LLC **(B)** and A549 cells **(C)** are depicted. **(D-E)** LLC and A549 cells were treated with DMF solvent control, CDDP (100μM), OGD/R30min, O+C, or thapsigargin (2μM) for 30 min, then cells were harvested, and proteins were detected by western blot. Densitometry data of p-eIF2α/ eIF2α in LLC **(D)** and A549 cells **(E)** are depicted. **(F)** LLC and A549 cells were treated as in A and B for 24 h, and then, the representative confocal images show the HMGB1 distribution in cells. All the data were from three independent experiments. Data are reported as the mean ± SEM, and statistical analyses were performed with one-way ANOVA followed by the Dunnett post hoc test. *P < 0.05, **P < 0.01, ***P< 0.001 and ****P< 0.0001.


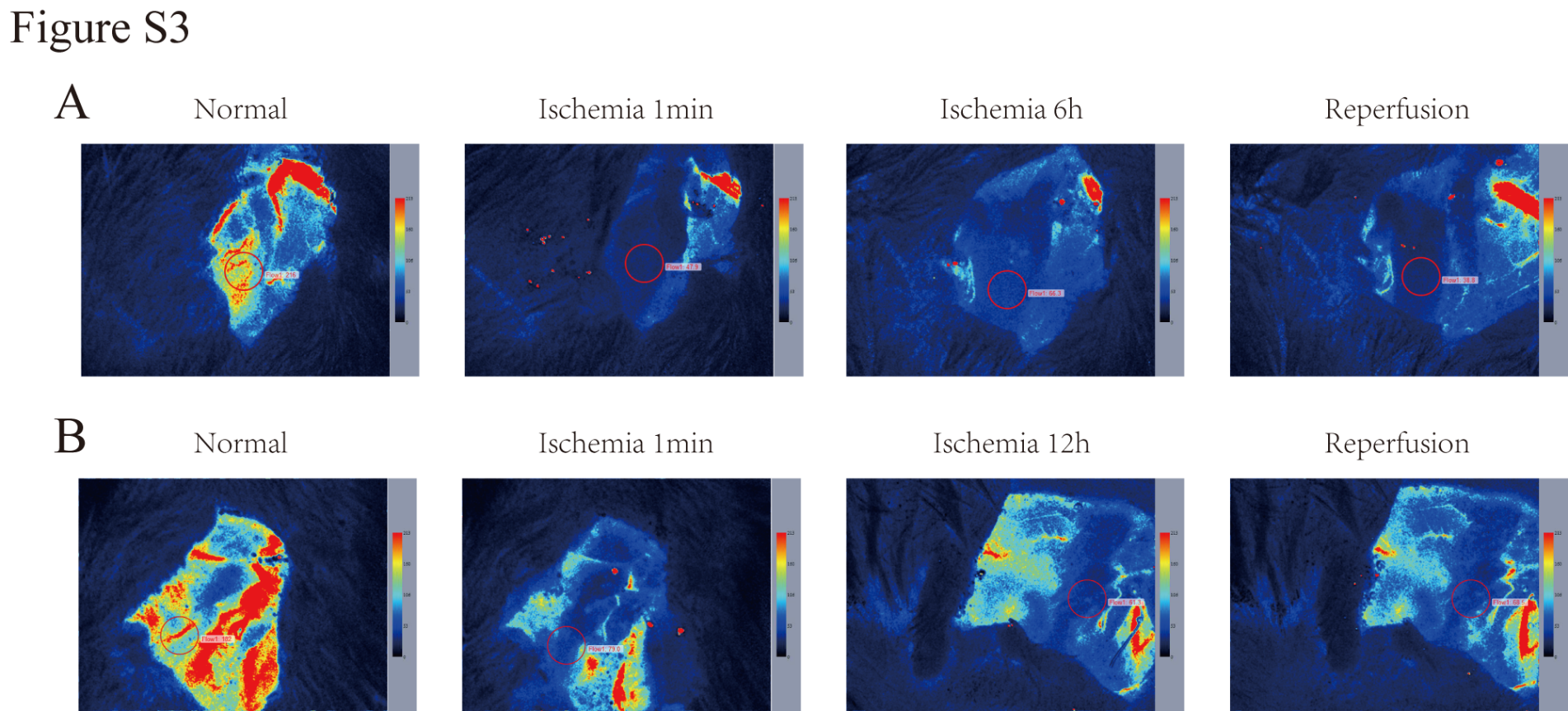


**Figure S3**. Representative laser speckle contrast images of inguinal tumor tissues before and during I/R. Ischemic 6 hours **(A)** and 12 hours **(B)**.


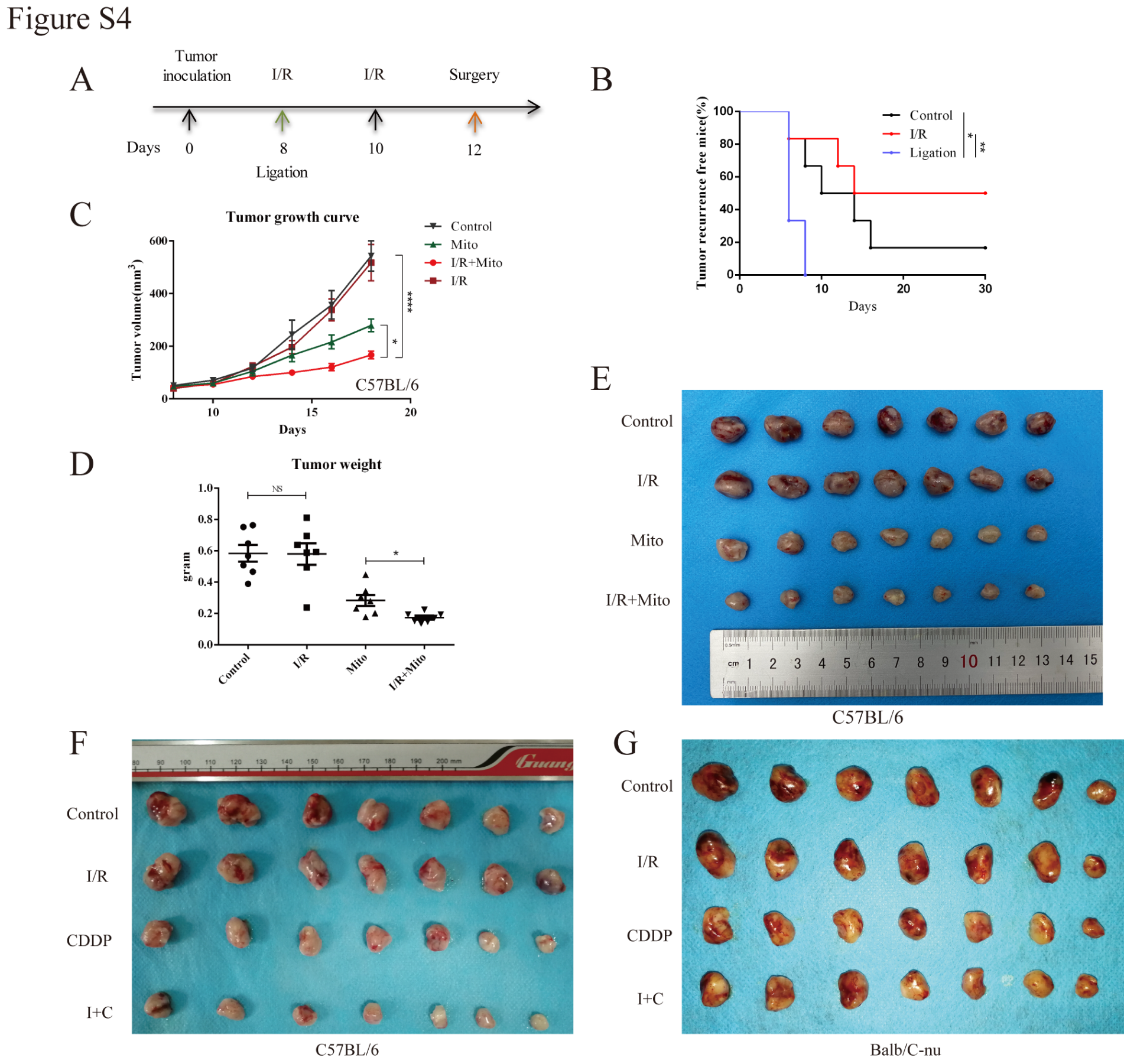


**Figure S4**. I/R combined with non-immunogenic chemotherapeutic drugs inhibit tumor growth. (A and B) A total of 5×10^5^ cells were inoculated subcutaneously into the right groin of C57BL/6 mice. On days 8, the right superficial abdominal artery of the mice was completely blocked or blocked for 90 min and then allowed to reperfuse. On day 12, all groin tumors and right inguinal lymph nodes were removed by surgery **(A)**. The tumor recurrence-free survival of the C57BL/6 mice after surgery, n=6 for each group **(B)**. **(C-E)** A total of 5×10^5^ cells were inoculated subcutaneously into the right groin of C57BL/6 mice. On days 8 and 10, the right superficial abdominal artery of the mice was blocked for 90 min and then allowed to reperfuse. On days 8, 10 and 13, 2.5 mg/kg Mitomycin C or PBS was injected intraperitoneally. n=7 for each group. The tumor growth curve **(C)**, plot of the tumor weight **(D)** and excised tumor photograph **(E)** of C57BL/6 mice after treated by I/R plus Mitomycin C. **(F)** On day 18, the excised tumor photograph of C57BL/6 mice after treated by I+C. **(G)** On day 18, the excised tumor photograph of Balb/C-nu mice after treated by I+C. Data are reported as the mean ± SEM, and statistical analyses were performed with one-way ANOVA followed by the Sidak post hoc test (D) and two-way ANOVA (C), The tumor incidence in the vaccination experiments was analyzed by means of the log-rank test. *P < 0.05, and ****P < 0.0001.


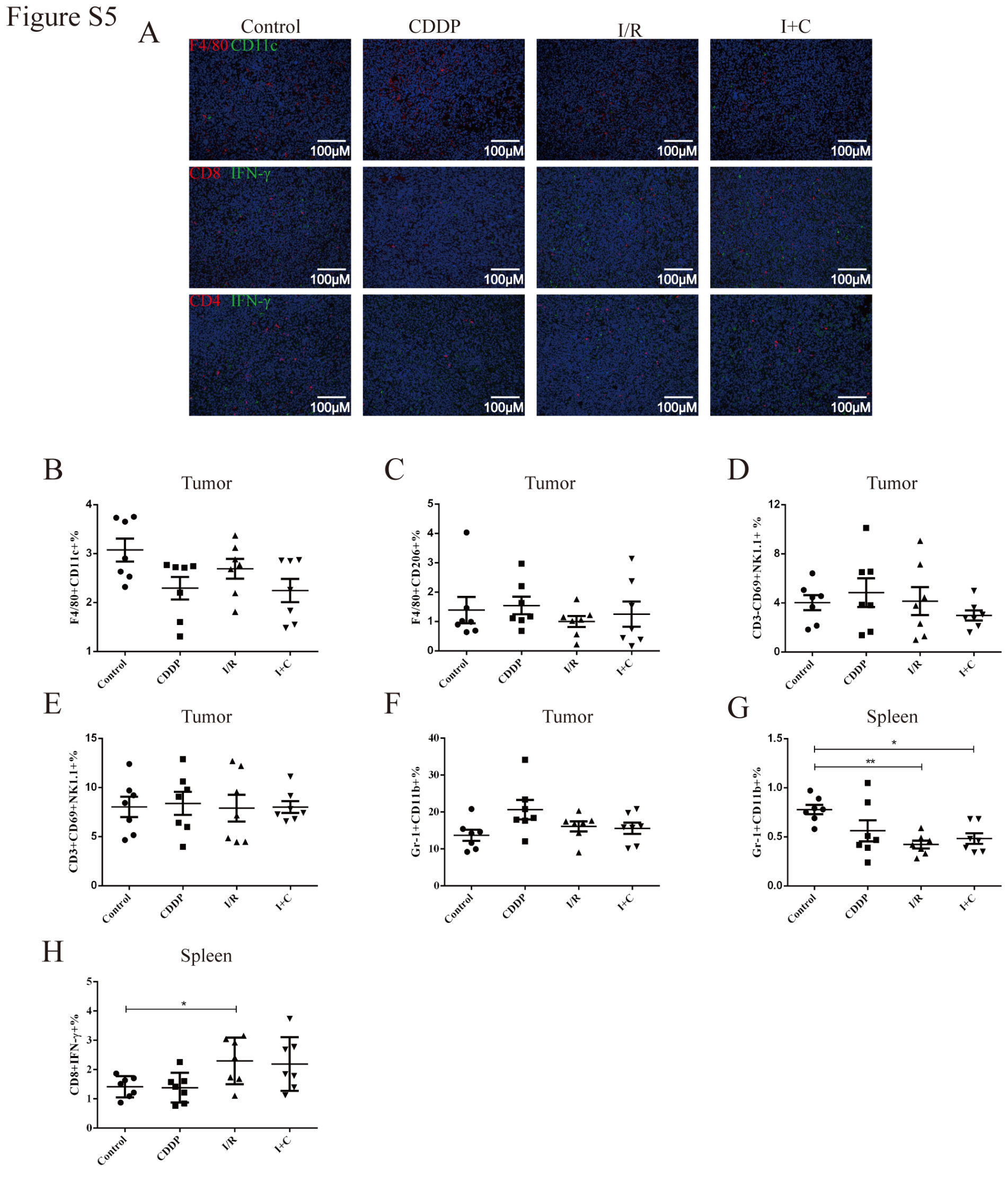


**Figure S5**. Immune cell subsets in the tumor and spleen. **(A)** Representative immunofluorescence images show the expression of CD8 (red), IFN-γ (greed), CD4 (red), F4/80 (red) and CD11c (green) in LLC tumors after different treatment. F4/80+CD11c+ cells **(B)**, and F4/80+CD206+ cells **(C)**, CD3-CD69+NK1.1+ cells **(D),** CD3-CD69+NK1.1+ cells **(E)**, Gr-1+CD11b+ cells **(F)** in the tumor were determined after gating on the live CD45+ cells. Gr-1+CD11b+ cells **(G),** CD8+IFN-γ+ cells **(H)** in the spleen were determined after gating on the live cells. N=7 for each group, each symbol represents a single mouse. Data are reported as the mean ± SEM, and statistical analyses were performed with one-way ANOVA followed by the Dunnett post hoc test. *P < 0.05, **P< 0.01.
