# Supplementary material for: Ischemia and reperfusion injury combined with cisplatin induces immunogenic cell death in lung cancer cells
Source: Cell Death Dis. 2022 Sep 3;13(9):764. doi: 10.1038/s41419-022-05176-y (PMC9440929; doi:10.1038/s41419-022-05176-y)
Supplement: Supplementary file 2 — Supplementary Materials for western blot [file 41419_2022_5176_MOESM2_ESM.docx]

Figure 2E

LLC p-eIF2α


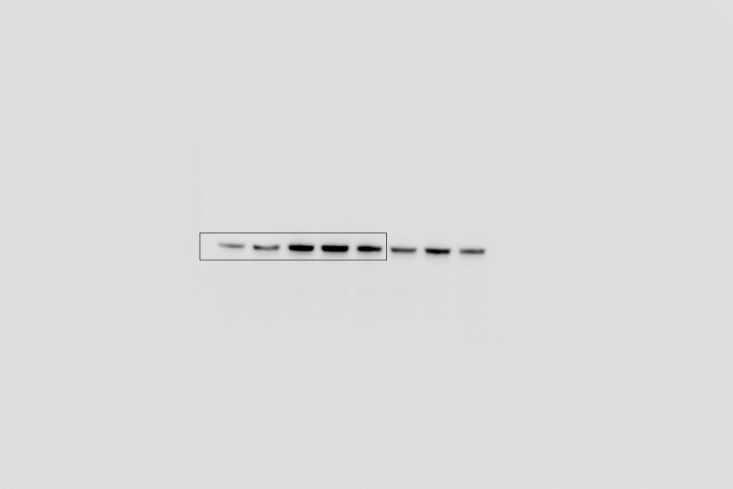


LLC eIF2α


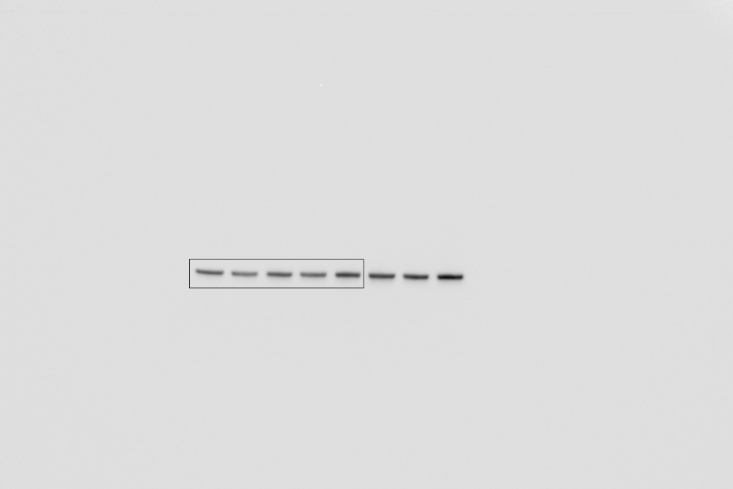


LLC actin


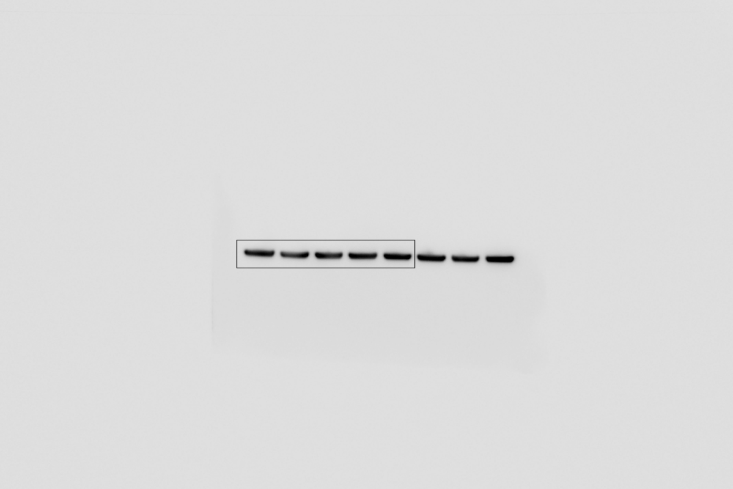


A549 p-eIF2α


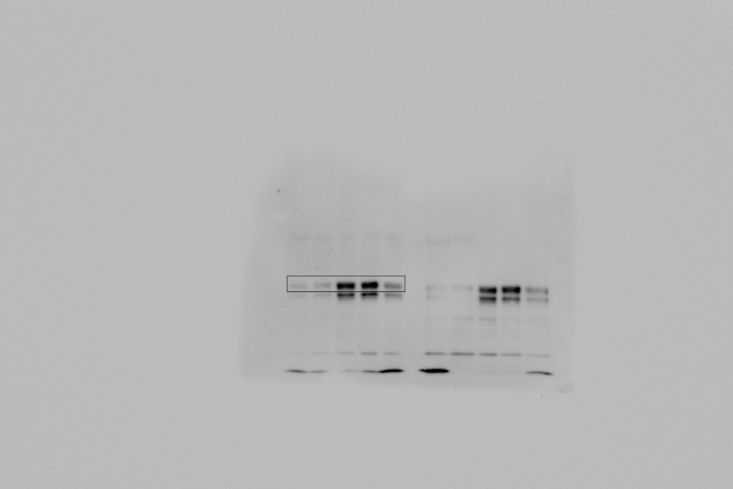


A549 eIF2α


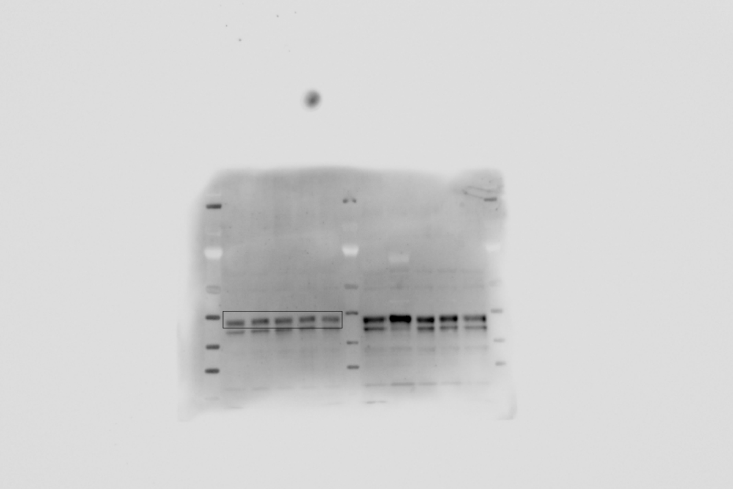


A549 actin


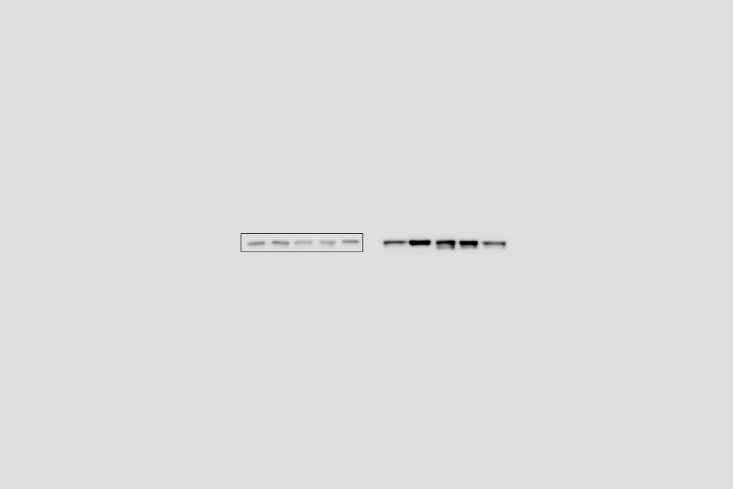


Figure 4A

p-eIF2α


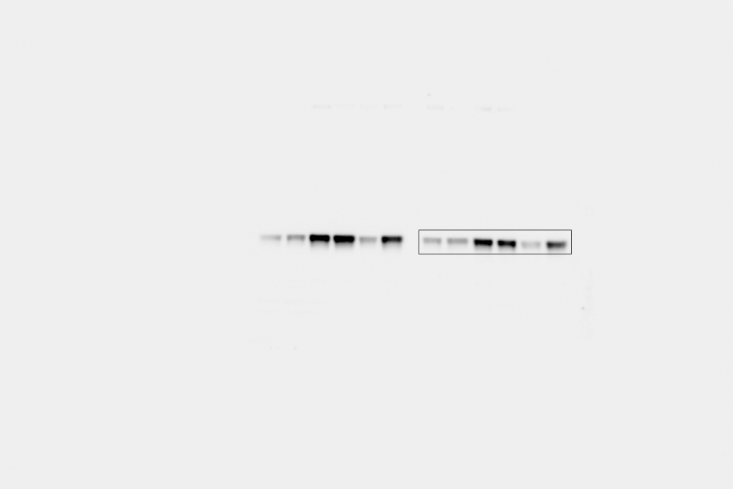


eIF2α


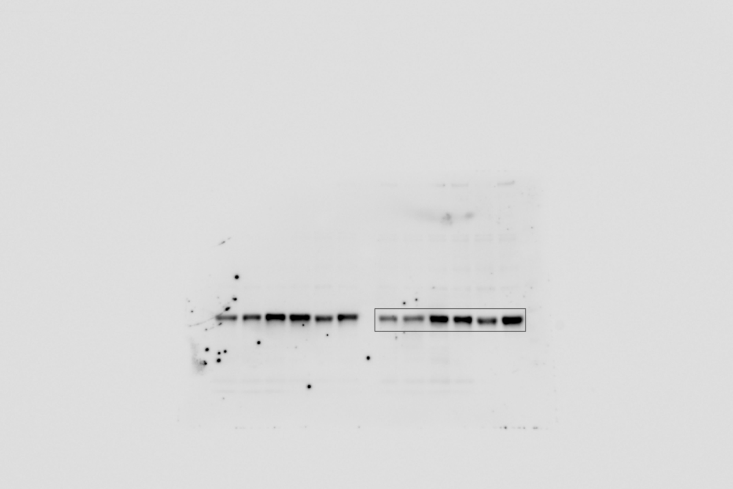


Actin


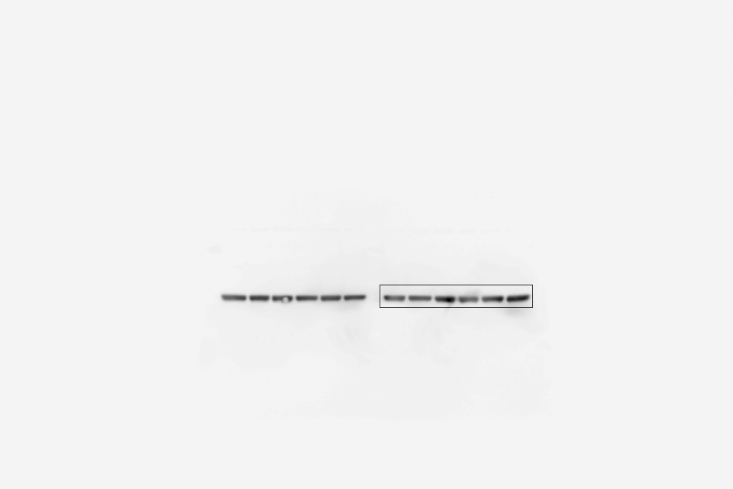


Figure 4C

PERK


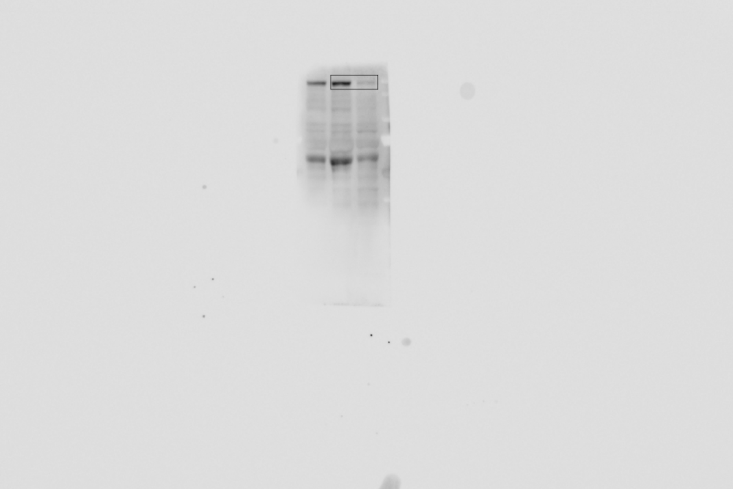


p-eIF2α


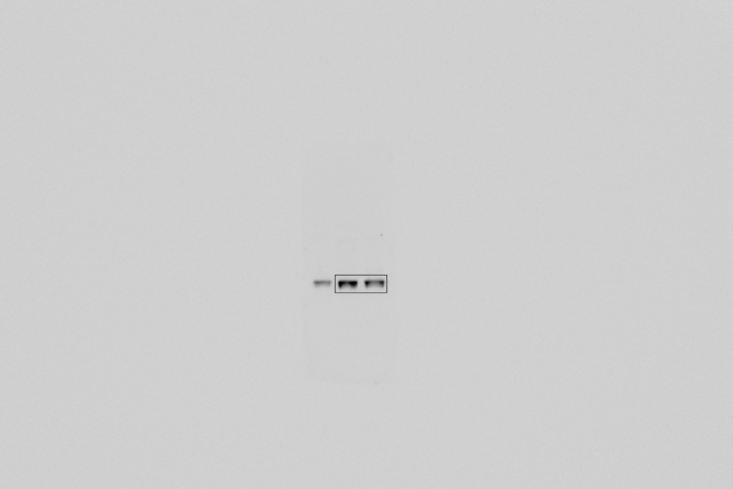


eIF2α


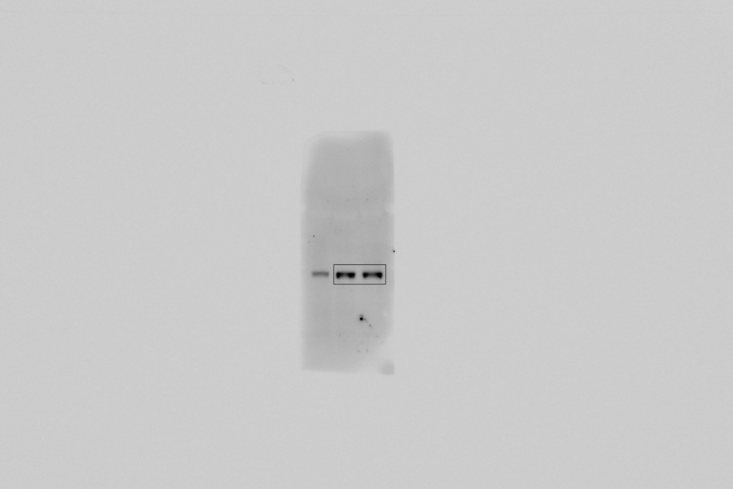


Actin


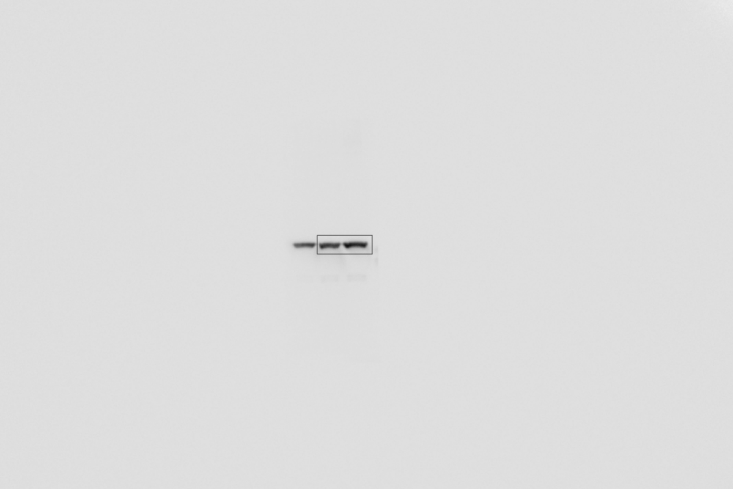


Figure 5I

p-eIF2α


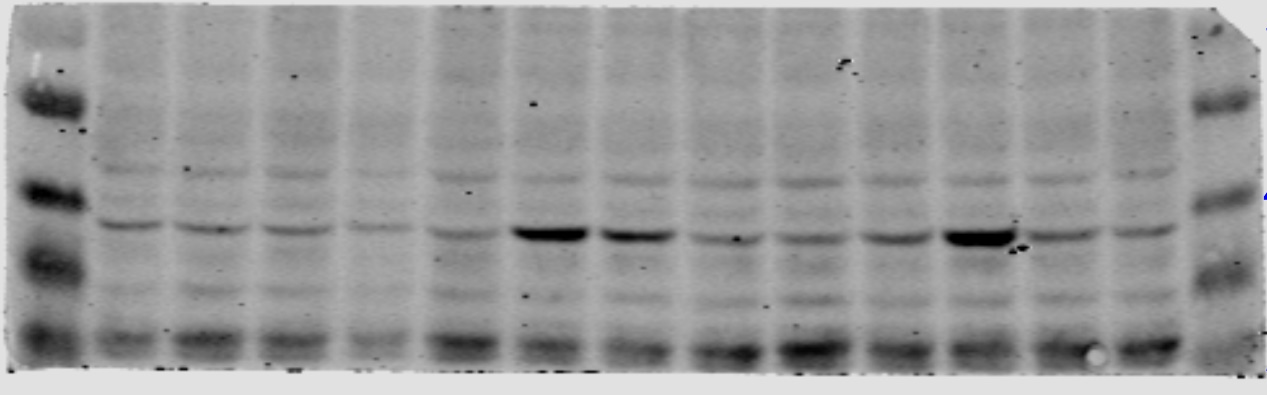


eIF2α


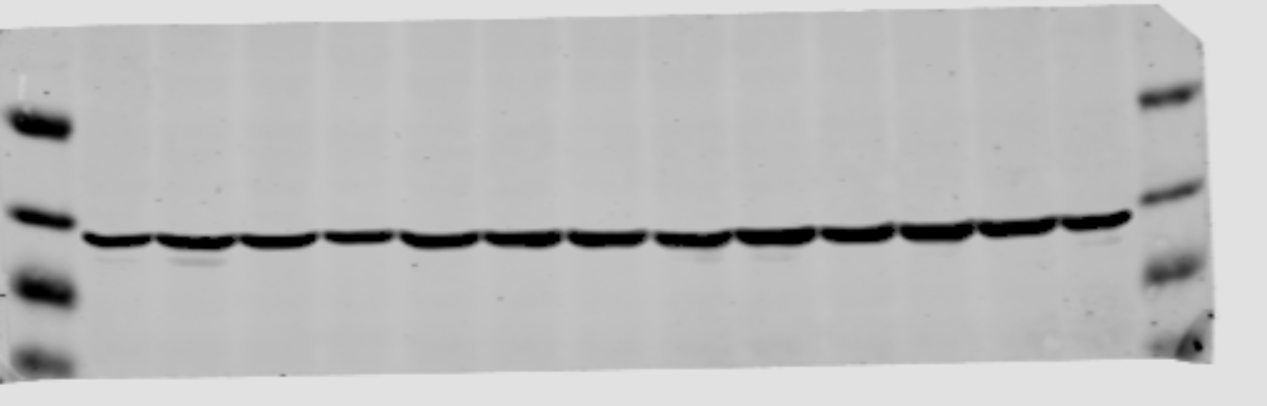


Actin


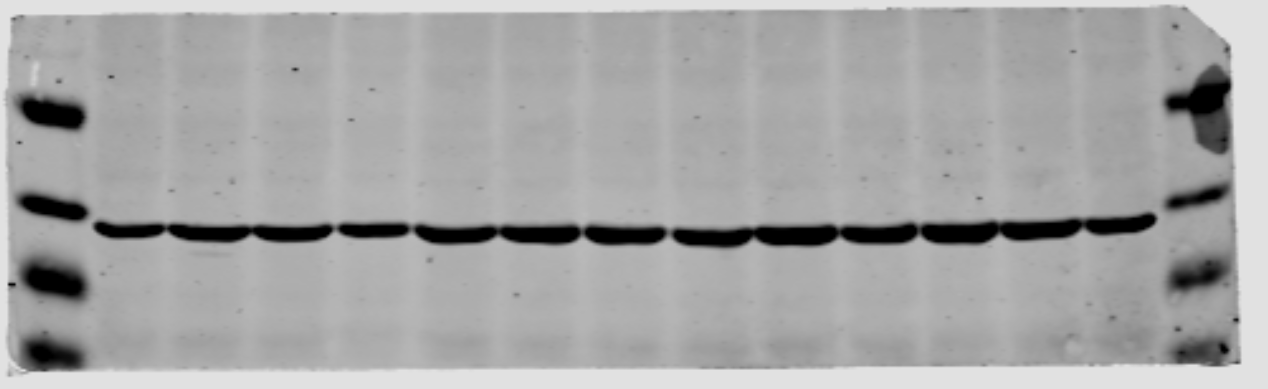


Figure S2A

CRT





Actin
